# Timescale separation and models of symbiosis: state space reduction, multiple attractors and initialization

**DOI:** 10.1093/conphys/coac026

**Published:** 2022-05-10

**Authors:** Ferdinand Pfab, Alexandra Lynne Brown, A Raine Detmer, Ethan C Baxter, Holly V Moeller, Ross Cunning, Roger M Nisbet

**Affiliations:** 1 Department of Ecology, Evolution and Marine Biology, University of California, Santa Barbara, CA 93106, USA; 2 Daniel P. Haerther Center for Conservation and Research, G. Shedd Aquarium, 1200 S. DuSable Lake Shore Drive, Chicago, IL 60605, USA

## Abstract

Dynamic Energy Budget models relate whole organism processes such as growth, reproduction and mortality to suborganismal metabolic processes. Much of their potential derives from extensions of the formalism to describe the exchange of metabolic products between organisms or organs within a single organism, for example the mutualism between corals and their symbionts. Without model simplification, such models are at risk of becoming parameter-rich and hence impractical. One natural simplification is to assume that some metabolic processes act on ‘fast’ timescales relative to others. A common strategy for formulating such models is to assume that ‘fast’ processes equilibrate immediately, while ‘slow’ processes are described by ordinary differential equations. This strategy can bring a subtlety with it. What if there are multiple, interdependent fast processes that have multiple equilibria, so that additional information is needed to unambiguously specify the model dynamics? This situation can easily arise in contexts where an organism or community can persist in a ‘healthy’ or an ‘unhealthy’ state with abrupt transitions between states possible. To approach this issue, we offer the following: (a) a method to unambiguously complete implicitly defined models by adding hypothetical ‘fast’ state variables; (b) an approach for minimizing the number of additional state variables in such models, which can simplify the numerical analysis and give insights into the model dynamics; and (c) some implications of the new approach that are of practical importance for model dynamics, e.g. on the bistability of flux dynamics and the effect of different initialization choices on model outcomes. To demonstrate those principles, we use a simplified model for root-shoot dynamics of plants and a related model for the interactions between corals and endosymbiotic algae that describes coral bleaching and recovery.

## Teaser

Dynamic Energy Budget models can describe species interactions that involve the exchange of metabolic products between organisms, for example the mutualism between corals and their symbionts. The paper describes a new mathematical approach for the analysis of such models including an application to coral bleaching.

## 1 Introduction

Metabolic models are used to describe physiological processes in living organisms. They range in mathematical and computational complexity from high-dimensional, parameter-rich, systems biology models used in biomedical applications to low-dimensional models with many fewer parameters exemplified by models based on Dynamic Energy Budget (DEB) theory (Kooijman, 2010). Some of Kooijman’s pioneering work in this field was motivated by challenges in environmental management, notably in ecotoxicology (Kooijman & Metz, 1984), with his first book subtitled *Theory and Applications in Ecotoxicology* (Kooijman, 1993). This focus remains important, e.g. (Murphy *et al.*, 2018), and subsequent research has greatly widened the scope of applications to many areas of conservation physiology, notably changes in a species’ ecological niche in response to environmental change (Kearney & Porter, 2020). Lavaud *et al.* (2021) offer an overview of such applications.

DEB models have been used in innovative research for nearly two decades to describe networks for the exchange of metabolic products between interacting organisms (Kooi *et al.*, 2004, Kooijman *et al.*, 2003), but this ecologically important area is still underdeveloped. The conceptual foundation within DEB theory for such models is the work by Kooijman (2001), which sketches links involving excretion fluxes from animal host and algal symbiont in corals and interactions between root and shoot in a plant. Subsequent models for the coral symbiosis and the interaction of roots and shoots in plants have been developed in Muller *et al.* (2009), Cunning *et al.* (2017), Ledder *et al.* (2020) and Schouten *et al.* (2020). These models can be applied to describe responses to environmental stress, e.g. coral bleaching (Cunning *et al.*, 2017, Eynaud *et al.*, 2011).

**Figure 1:**
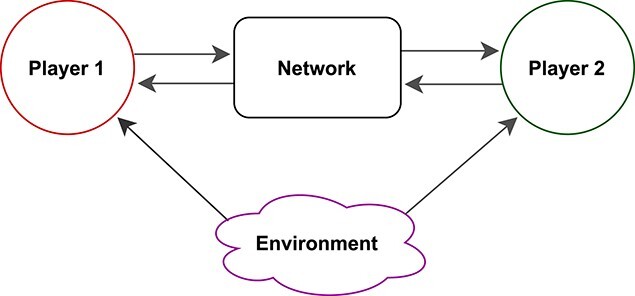
Schematic representation of a symbiotic interaction.

Mathematically, several of the cited models take the form of differential equations that involve a small number of state variables. For instance, the models in (Cunning *et al.*, 2017) and Ledder *et al.* (2020) describe two ‘players’ who exchange metabolic products that are surplus to their own needs. This interaction is described with a set of algebraic equations that characterize the flows of elemental matter within an interaction network; see schematic Fig. 1. The algebraic equations arise from the assumption that reactions and translocations within the network occur on a much faster timescale than changes in the state variables for the biomass of the interacting organisms. Therefore, the reactions and translocations can be assumed to equilibrate virtually immediately. This timescale separation allows for simpler model formulation with fewer parameters and can greatly improve numerical speed for simulations (a concern when using computer-intensive methods for parameter estimation).

Even conceptually simple networks typically involve many interconnected fluxes. As a consequence, such networks can have multiple equilibrium states. In those cases, unambiguous tracking of only slow variables is not possible without at minimum additional information about the state of the fast dynamic network. An example for such a situation is the earlier mentioned model for the growth of the root and the shoot of a plant by Ledder *et al.* (2020). This model does not only need information on the biomass of the two organelles, but it also needs to track the current rates at which root and shoot share nitrogen and carbon, respectively. The initialization of these rates can determine whether the system starts with either the root growing or the shoot growing. A similar example is the model for the interactions between a coral host and its endosymbiotic algae by Cunning *et al.* (2017). In this model, the initial fluxes can not only influence the initial growth phase but also determine whether the system reaches a healthy state with a high symbiont density or an unhealthy state where the coral loses its symbiont and starves eventually (coral bleaching). The work by Cunning *et al.* (2017) demonstrates how the issue of multiple fast equilibria in DEB models has been approached previously with a discrete-time scheme. In this scheme, the ambiguity of the model definition is resolved by adding state variables for the fast variables and using the state of the system at the preceding time step in the update step.

In the present paper, we propose a new approach for the issue of tracking the fast network. The new scheme adds an explicit continuous-time representation of the fast dynamics by introducing additional state variables. Those additional state variables buffer some connections in the network and in this way complete the definition of the fast dynamics. Among the advantages of this new method is that it relies on continuous time and thus allows simulating models with standard solvers for ordinary differential equations.

While this new approach can be used generally as a numerical approximation of unknown fast dynamics, we show how under certain circumstances it can be interpreted mechanistically. We further connect this new approach to previous work by showing that the continuous-time approach is a generalization of the discrete-time scheme used before.

Our approach for completing implicitly defined models is based on additional state variables (buffers) that interrupt cycles in the metabolic network. Naturally, it is possible to track all auxiliary variables, but often it is possible to only track some of them and still fully define the model. This reduces the dimensionality of the system, which can simplify model analysis. We will show that graph theory can be used to decide which variables to track and to find the minimal number of additional state variables that are needed to fully describe such systems.

The paper is structured as follows: Section 2 describes the key steps in our approach to timescale separation. In Sections 3 and 4 we use the new methods in analyses of the simple root–shoot model from Ledder *et al.* (2020) and the more complex model for the dynamics of corals with their endosymbiotic algae partners from Cunning *et al.* (2017). In Section 5 we discuss our findings and offer an outlook on wider areas of application, including modeling complex metabolic networks that are relevant to pressing environmental challenges.

## 2 Timescale separation in metabolic models

### 2.1 Cyclic and acyclic metabolic networks.

The Introduction highlighted dynamic bioenergetic models in which the state ${{X}\kern-6pt^{{}^{{}^{\rightarrow}}}}$ of interacting organisms (or of organs within a single organism) is described by a set of *differential* equations coupled to an auxiliary network of variables, ${{Z}\kern-6pt^{{}^{{}^{\rightarrow}}}}$. A general form for the differential equation for ${{X}\kern-6pt^{{}^{{}^{\rightarrow}}}}$ is

(1) \begin{align*}& \begin{aligned} \frac{d {{X}\kern-6pt^{{}^{{}^{\rightarrow}}}}}{dt}&=f({{X}\kern-6pt^{{}^{{}^{\rightarrow}}}},{{Z}\kern-6pt^{{}^{{}^{\rightarrow}}}}). \end{aligned} \end{align*}

The auxiliary variables ${{Z}\kern-6pt^{{}^{{}^{\rightarrow}}}}$ most commonly represent fluxes. To minimize abstraction, we refer to them as fluxes in the remainder of this section. Although the fluxes ${{Z}\kern-6pt^{{}^{{}^{\rightarrow}}}}$ may depend on the state variables, they are typically assumed to change on a sufficiently fast timescale that they can be replaced at any time by their equilibrium values (given the current state ${{X}\kern-6pt^{{}^{{}^{\rightarrow}}}}$). We shall on occasions refer to these value as ‘fast equilibrium’. A model is unambiguously defined in this way when the auxiliary variables can be expressed as simple functions of the state variables,

(2) \begin{align*}& \begin{aligned} {{Z}\kern-6pt^{{}^{{}^{\rightarrow}}}} &= g ( {{X}\kern-6pt^{{}^{{}^{\rightarrow}}}} ). \end{aligned} \end{align*}

**Figure 2:**
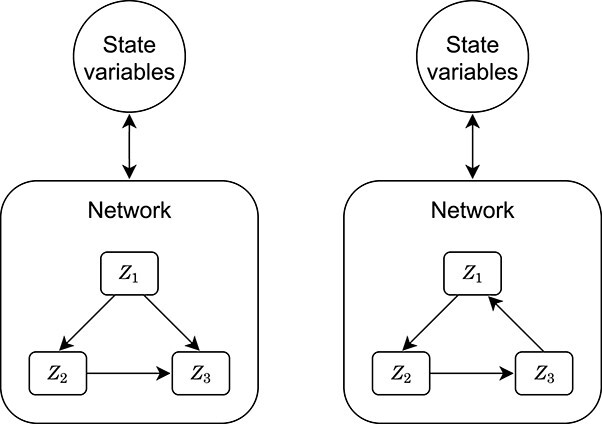
Networks for fluxes ${{Z}\kern-6pt^{{}^{{}^{\rightarrow}}}}$. Left: an acyclic graph, the model is defined uniquely. Right: a cyclic graph, the model is potentially not uniquely defined.

An example for such a system is shown on the left side of Fig. 2. In this example, knowledge of the state variables permits direct evaluation of $Z_{1}$, which in turn allows evaluation of $Z_{2}$ and $Z_{3}$.

Such sequential evaluation is not necessarily possible if the fluxes in the network depend on each other in a cyclic fashion (right side of Fig. 2). In this case, the fast equilibrium states are defined *implicitly* as solutions of an equation (almost always non-linear) of the form

(3) \begin{align*}& \begin{aligned} {{Z}\kern-6pt^{{}^{{}^{\rightarrow}}}} &= g ( {{X}\kern-6pt^{{}^{{}^{\rightarrow}}}}, {{Z}\kern-6pt^{{}^{{}^{\rightarrow}}}} ). \end{aligned} \end{align*}

With an implicitly defined network, it is possible that multiple values of ${{Z}\kern-6pt^{{}^{{}^{\rightarrow}}}}$ satisfy the equilibrium condition. In this case, additional assumptions on fast dynamics are needed to fully specify the model. This is not a mere mathematical nicety: the two case studies in subsequent sections will show that the initial state of a network will frequently have lasting impact on the long-term (slow) dynamics of the full system. In short, to unambiguously define our models, we need to specify fast dynamics.

### 2.2 Differential equations for fast and slow dynamics

To complete models with implicitly defined auxiliary networks, we set up hypothetical fast dynamics that mimic the (generally unknown) actual fast dynamics. Those hypothetical fast dynamics should satisfy two conditions. First, at their equilibrium they should satisfy the model specification ${{Z}\kern-6pt^{{}^{{}^{\rightarrow}}}} = g ( {{X}\kern-6pt^{{}^{{}^{\rightarrow}}}}, {{Z}\kern-6pt^{{}^{{}^{\rightarrow}}}} )$ as stated in equation (3). Second, each flux $Z_i$ should approach its ‘target’ $g_i ( {{X}\kern-6pt^{{}^{{}^{\rightarrow}}}}, {{Z}\kern-6pt^{{}^{{}^{\rightarrow}}}} )$. Arguably, the simplest solution for these dynamics is to let each flux decay exponentially towards its target. We therefore propose to replace the implicit equation for ${{Z}\kern-6pt^{{}^{{}^{\rightarrow}}}}$ with the dynamical system

(4) \begin{align*}& \begin{aligned} \frac{d Z_i}{dt} &= \lambda_i ( g_i({{X}\kern-6pt^{{}^{{}^{\rightarrow}}}},{{Z}\kern-6pt^{{}^{{}^{\rightarrow}}}}) - Z_i ), \end{aligned} \end{align*}

where the set of parameters {$\lambda _i$} determines how quickly the fluxes approach the fast equilibrium. When the values for the $\lambda _i$ are high, the fluxes typically track their targets tightly. The differential equations for the auxiliary state variables ${{Z}\kern-6pt^{{}^{{}^{\rightarrow}}}}$ and the original state variables ${{X}\kern-6pt^{{}^{{}^{\rightarrow}}}}$ now fully define the model dynamics, given initial values for both ${{X}\kern-6pt^{{}^{{}^{\rightarrow}}}}$ and ${{Z}\kern-6pt^{{}^{{}^{\rightarrow}}}}$.

The anticipated properties of our scheme for model completion are supported by a mathematical result—Tikhonov’s theorem (Klonowski, 1983, Tikhonov, 1952). Expressed informally, the theorem states that in systems with fast and slow variables the fast dynamics typically track an isolated fast equilibrium after some initial transient (i.e. the auxiliary variables are sticking close to their previous state). Appendix A gives a precise statement of the theorem together with three conditions for it to hold. Appendix B outlines in detail the reasoning that connects the theorem to our differential equation scheme and also shows that the current approach is asymptotically a generalized form of the discrete time method used in a previous work (Cunning *et al.*, 2017).

Our choice of equations for fast dynamics was motivated by mathematical requirements and took no account of the physiological/biochemical nature of the networks. There is no reason to believe that they represent the ‘real’ underlying fast dynamics that are generally unknown. In some cases, however, the differential equations we introduced can plausibly represent original fast dynamics. The root–shoot model is such a case. We work out the details for this example in the section for this model. Generally, our numerical scheme emerges when the nodes of the auxiliary network are assumed to act as ‘buffers’ that delay inputs within the network. Those buffers then represent the state of the network. Assume the following fast network. The state of each part $i$ of a network is described by a buffer $E_i$ that feeds into the network at a rate $\lambda _i E_i$. The input of the buffers is given by $g_i({{X}\kern-6pt^{{}^{{}^{\rightarrow}}}},\Lambda {{E}\kern-6pt^{{}^{{}^{\rightarrow}}}})$ (where $\Lambda $ is a diagonal matrix with the $\lambda _i$ on its diagonal). This gives us the differential equation for the buffers:

(5) \begin{align*}& \begin{aligned} \frac{d {{E}\kern-6pt^{{}^{{}^{\rightarrow}}}}}{dt} &= g({{X}\kern-6pt^{{}^{{}^{\rightarrow}}}}, \Lambda {{E}\kern-6pt^{{}^{{}^{\rightarrow}}}}) - \Lambda {{E}\kern-6pt^{{}^{{}^{\rightarrow}}}}. \end{aligned} \end{align*}

Letting ${{Z}\kern-6pt^{{}^{{}^{\rightarrow}}}}=\Lambda {{E}\kern-6pt^{{}^{{}^{\rightarrow}}}}$ we find

(6) \begin{align*}& \begin{aligned} \frac{d {{Z}\kern-6pt^{{}^{{}^{\rightarrow}}}}}{dt} &= \frac{d \Lambda {{E}\kern-6pt^{{}^{{}^{\rightarrow}}}}}{dt} &=\Lambda ( g({{X}\kern-6pt^{{}^{{}^{\rightarrow}}}},{{Z}\kern-6pt^{{}^{{}^{\rightarrow}}}}) -{{Z}\kern-6pt^{{}^{{}^{\rightarrow}}}}). \end{aligned} \end{align*}

This is equivalent to the implementation described earlier, equation (4).

### 2.3 Reducing the dimensionality of the fast network

The natural implementation of our approach is to track all auxiliary variables by additional state variables. This increases the dimensionality of the system by the number of auxiliary variables. However, often the auxiliary network can be already fully described with less state variables. This can be useful for analytical and numerical model analysis, particularly because auxiliary variables need to be initialized and this initialization then in turn can determine the long-term (slow) dynamics of the system. To reduce the dimensionality of the auxiliary fast dynamics, we find combinations of auxiliary variables through which the rest of the auxiliary network can be calculated.

Biologically, this state space reduction corresponds to implementing a time scaling argument within the fast dynamics—some processes are on a fast timescale and some on a very fast timescale. The fast processes are tracked with differential equations, and the very fast processes are assumed to equilibrate instantaneously and are calculated directly.

When there is no biological intuition on the relative speed at which the different fast processes act, graph theory can be used to find low-dimensional representations of the network. For this purpose, we represent the interactions of the model fluxes with a directed graph as shown in Fig. 2. The nodes of the graph represent the fluxes, and the arrows indicate the connections between the fluxes. An arrow from flux $Z_i$ to flux $Z_j$ indicates that $Z_i$ appears in the function for calculating $Z_j$. When this graph is acyclic, the model specification readily defines the fluxes uniquely and no additional state variables are needed to simulate the system (left side of Fig. 2). On the other hand, when this graph is cyclic, the model equations may be satisfied for different combinations of the fluxes (right side of Fig. 2). Tracking a flux by an additional state variable removes this flux from the network of algebraic equations because this flux does not equilibrate instantaneously anymore. This interrupts all cycles in which the flux is involved. Thus, in order to fully define the system, we need to find combinations of fluxes that, when expressed by state variables, interrupt all cycles in the graph. In the example on the right side of Fig. 2, tracking any of the fluxes with a state variable is enough to interrupt all cycles and uniquely define the system. The remaining fluxes can be calculated one by one from the value of this flux (and the slow dynamics, which have not been specified in the figure). The process is formally set out in Appendix C.

The *Mathematica* code in the supplementary material demonstrates how to identify whether a combination of fast state variables leads to an acyclic graph and how to find the minimal number of buffers that renders the fast dynamic network acyclic and defines the model uniquely.

In the following sections, we will illustrate those concepts with examples.

**Figure 3:**
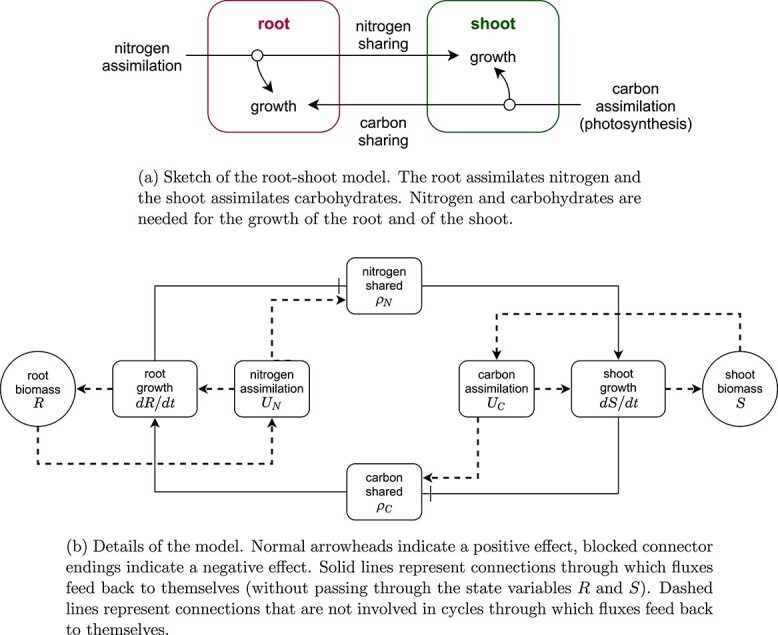
The root–shoot model.

## 3 The root–shoot model

This is a simplified version of the model presented in Ledder *et al.* (2020). The model is sketched in Fig. 3a. It describes how the root and the shoot of a plant interact. The root provides nitrogen and the shoot provides reduced carbon. Both components need nitrogen and carbon to grow. The root shares surplus nitrogen not used with the shoot, and the shoot shares surplus carbon with the root.

### 3.1 Model formulation

A detailed diagram of the model is shown in Fig. 3b. The state variables, fluxes and parameters are summarized in Table 1.

**Table 1:** Model parts of the root-shoot model

State variables		
Symbol	Description	Typical units
$R$	Root biomass (in carbon)	mol C
$S$	Shoot biomass (in carbon)	mol C
Fluxes		
Symbol	Description	Typical units
$U_N$	Nitrogen assimilated by root	mol N d$^{-1}$
$U_C$	Carbon assimilated by shoot	mol C d$^{-1}$
$\rho _N$	Nitrogen shared by root	mol N d$^{-1}$
$\rho _C$	Carbon shared by root	mol C d$^{-1}$
Parameters		
Symbol	Description	Typical units
$\alpha _N$	Nitrogen assimilation rate of root	mol N mol C$^{-1}$ d$^{-1}$
$\alpha _C$	Photosynthesis rate of shoot	mol C mol C$^{-1}$ d$^{-1}$
$\eta _R$	Nitrogen–carbon ratio of root	mol N mol C$^{-1}$
$\eta _S$	Nitrogen–carbon ratio of shoot	mol N mol C$^{-1}$

The equations for the root–shoot model are as follows. The assimilation of nitrogen by the root and the assimilation of carbohydrates (short:carbon) by the shoot are

(7) \begin{align*}& \begin{aligned} U_N &= \alpha_N R \\ U_C &= \alpha_C S, \end{aligned} \end{align*}

where the parameters $\alpha _N$ and $\alpha _C$ describe the rates at which root and shoot produce nitrogen and carbon, respectively. The version of the model we describe here assumes that growth of each organ is limited by the more limiting resource, i.e. growth is governed by a minimal synthesizing unit (SU) (Ledder *et al.*, 2020). Growth of root and shoot is given by

(8) \begin{align*}& \begin{aligned} \frac{dR}{dt} &= \min(\rho_C, \eta_R^{-1} U_N) \\ \frac{dS}{dt} &= \min(U_C, \eta_S^{-1} \rho_N), \end{aligned} \end{align*}

where the parameters $\eta _R^{-1}$ and $\eta _S^{-1}$ describe the N:C ratio in root and shoot biomass and $\rho _N$ and $\rho _C$ are the rates at which nitrogen and carbon are shared, respectively. In the original model formulation, the nutrient sharing rates are defined by the difference of assimilation and use, $\rho _N = g_{\rho _N}$ and $\rho _C = g_{\rho _C}$, where

(9) \begin{align*}& \begin{aligned} g_{\rho_N} &= U_N - \eta_R \frac{dR}{dt} \\ g_{\rho_C} &= U_C - \frac{dS}{dt}. \end{aligned} \end{align*}

### 3.2 Multiple solutions of flux combinations

**Figure 4:**
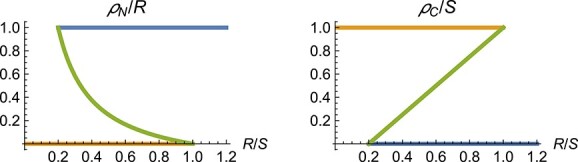
Combinations of the surplus fluxes $\rho _N / R$ and $\rho _C / S$ that satisfy the model equations in dependence of the root–shoot ratio $R/S$. Parameters: $\alpha _N=1$, $\alpha _C=1$, $\eta _R=1$ and $\eta _S=0.2$. The different colors indicate which branches belong together in the left panel and the right panel.

The model as defined above does not always define all fluxes uniquely. Given values for $R$ and $S$, the model equations can be satisfied for different combinations of the surplus sharing fluxes $\rho _N$ and $\rho _C$ (the auxiliary network). Figure 4 shows solutions for the surplus sharing fluxes as functions of the root–shoot ratio $R/S$. An analogous plot is shown in Fig. 3 of Ledder *et al.* (2020).

The reason multiple flux combinations can satisfy the original model equations is that the model fluxes for surplus sharing and growth form a closed loop that feeds back to itself, as shown in Fig. 3b. The loop can be described as follows. When the root grows, it shares less nitrogen, thus the shoot grows less (or not at all as in our example) and shares more carbon, reinforcing continuous growth of the root. In the other way around, when the shoot grows, it shares less carbon, thus the root grows less (or not at all as in our example) and shares more nitrogen, and shoot growth is reinforced. In the simplest form, this loop can be seen by expressing the two fluxes for surplus sharing as functions of each other by plugging in the equations for the growth rates of root and shoot,

(10) \begin{align*}& \begin{aligned} \rho_N &= U_N - \eta_R \frac{dR}{dt} \\ &= U_N - \eta_R \min(\rho_C, \eta_R^{-1} U_N)\\ \rho_C &= U_C - \frac{dS}{dt} \\ &= U_C - \min(U_C, \eta_S^{-1} \rho_N). \end{aligned} \end{align*}

**Figure 5:**
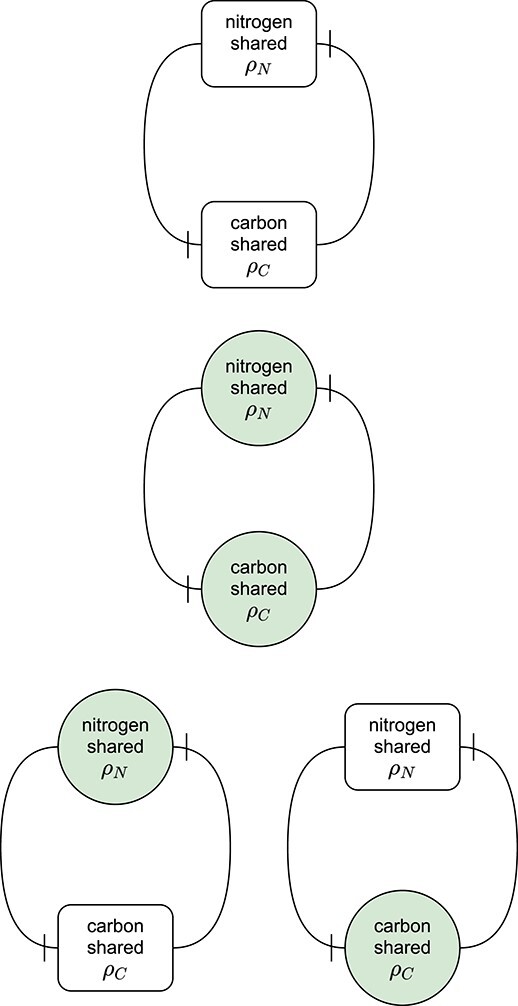
The surplus sharing fluxes $\rho _N$ and $\rho _C$ form a closed cycle in the root–shoot model. Blocked connector endings indicate a negative effect. Boxes indicate direct algebraic equations; green circles indicate fast state variables that buffer inputs and outputs. Both fluxes have a (negative) effect on the other flux. The more nitrogen the root shares, the more the shoot can grow, which in turn shares less carbon because its surplus is smaller. In the same way, a bigger surplus of carbon results in a smaller surplus of nitrogen. Top row: all interactions are direct, the model is not uniquely defined. All other rows introduce state variables that define the model uniquely. Middle row: state variables for $\rho _N$ and $\rho _C$. Bottom row: state variables for $\rho _N$ only (left) and for $\rho _C$ only (right).

This loop is schematized in Fig. 5 (top row).

### 3.3 Adding state variables to the model to obtain a uniquely defined system and simulate the model

As shown in Fig. 4, the network of auxiliary variables (the rates at which nitrogen and carbon are shared) can have up to three solutions for a given root-to-shoot ratio. Additional model assumptions are needed to simulate the system. Here we show how to unambiguously define the system using the approach outlined in Section 2. The method bases on adding state variables for the surplus sharing rates to the model equations so that the cycles within the auxiliary network are interrupted.

To derive the method mechanistically for the present example, we add buffers that gather the fluxes of nitrogen and carbon shared by the root and the shoot.

Nitrogen that is shared by the root is first captured in a buffer $E_{\rho _N}$. The input rate to the buffer is the rate at which the root produces surplus nitrogen, $g_{\rho _N}$. Additionally, we assume that the buffer empties at a rate $\lambda _{\rho _N} E_{\rho _N}$ into the growth SU for the shoot. This means the buffer changes according to

(11) \begin{align*}& \begin{aligned} \frac{d E_{\rho_N}}{dt} &= g_{\rho_N} - \lambda_{\rho_N} E_{\rho_N}. \end{aligned} \end{align*}

When the buffer is at equilibrium, $d E_{\rho _N}/dt=0$, and therefore $\lambda _{\rho _N} E_{\rho _N} = g_{\rho _N}$. Thus, in accordance with the model definition, we can identify $\rho _N=\lambda _{\rho _N} E_{\rho _N}$.

This rate $\rho _N$ at which nitrogen is shared can be expressed directly by the differential equation

(12) \begin{align*}& \begin{aligned} \frac{d \rho_N}{dt} &= \frac{d \lambda_{\rho_N} E_{\rho_N}}{dt} = \lambda_{\rho_N} (g_{\rho_N} - \rho_N ). \end{aligned} \end{align*}

The rate nitrogen is shared $\rho _N$ obviously follows its equilibrium $g_{\rho _N}$ (assuming the other state variables are constant). The speed at which the tracks its equilibrium is given by the parameter $\lambda _{\rho _N}$.

In the same way, we can introduce a differential equation for the rate at which carbon is shared, $\rho _C$,

(13) \begin{align*}& \begin{aligned} \frac{d \rho_C}{dt} &= \lambda_{\rho_C} ( g_{\rho_C} - \rho_C ). \end{aligned} \end{align*}

The model is now fully defined as a system of four differential equations that track the states of the biomass of root and shoot $R$ and $S$, and the rate at which nitrogen and carbon is shared, $\rho _N$ and $\rho _C$. This choice of additional state variables is shown in the bottom row of Fig. 5.

**Figure 6:**
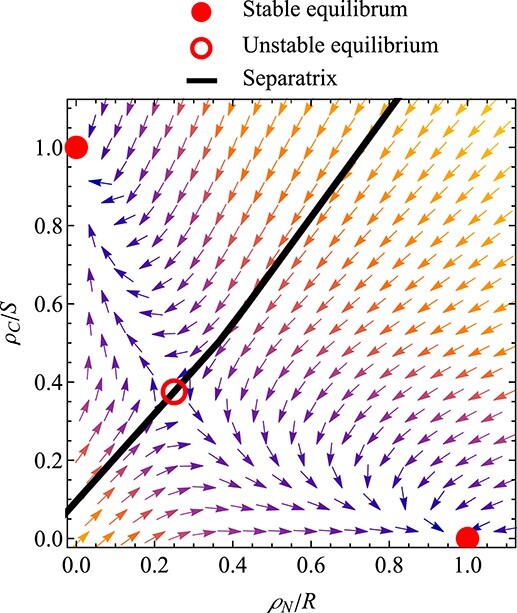
Fast dynamics of the root–shoot model, implemented with additional state variables for the shared surplus of nitrogen and carbon, $\rho _N/R$ and $\rho _C/S$. Biomass of root and shoot is kept constant. In the full system (with changing biomasses), this phase plane would change as the slow state variables evolve. The phase plane shown here reveals bistability of the fast dynamics. The system has three equilibria. The two outer (stable) equilibria correspond to states where $\rho _N/R$ is high and where $\rho _C/S$ is high (the other surplus sharing rate being zero). The equilibria characterize states where only the root growing and only the shoot growing. The two basins of attraction are separated by an unstable equilibrium and a separatrix. Brighter colors for the vectors indicate stronger attraction. Parameters: $\alpha _N=1$, $\alpha _C=1$, $\eta _R=1$, $\eta _S=0.2$, $\eta _S=0.2$, $\lambda _{\rho _N}=10$ and $\lambda _{\rho _C}=10$. Root and shoot biomass are fixed: $R=1$ and $S=2$.

A phase plane for the fast buffer dynamics is shown in Fig. 6. The simulations assume that the biomass of root and shoot are kept constant (assuming buffer dynamics are much faster than biomass dynamics). The plot shows the bistability of the (fast) system: when starting with a high rate of nitrogen shared $\rho _N$, the system converges to an equilibrium with high $\rho _N$; when starting with a high rate of carbon shared $\rho _C$, the system converges to an equilibrium with high $\rho _C$. These two equilibria correspond to states where, respectively, only the root or only the shoot is growing.

**Figure 7:**
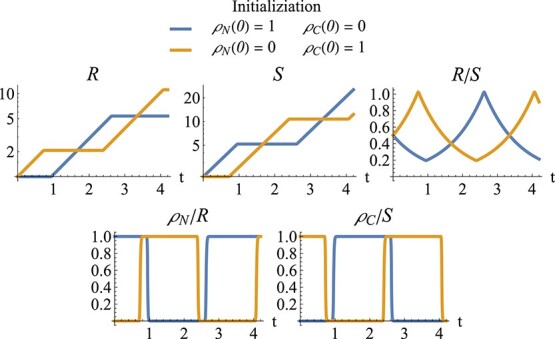
Simulations of root–shoot model, implemented with fast differential equations for the shared surplus of nitrogen and carbon, $\rho _N$ and $\rho _C$. From left to right and top to bottom: root biomass (log scale), shoot biomass (log scale), ratio of root biomass to shoot biomass, rate of nitrogen shared by root (relative to root biomass) and rate of carbon shared by shoot (relative to shoot biomass). Blue curves: simulations are started with a high rate of nitrogen shared and a low rate of carbon shared (bottom right of Fig. 6); the system starts with only the shoot growing. Orange curves: simulations are started with a high carbon buffer and a low nitrogen buffer (top left of Fig. 6); the system starts with only the shoot growing. Parameters as in Fig. 6. Initial values for root and shoot correspond to the (fixed) values for these state variables in Fig. 6: $R(0)=1$ and $S(0)=2$.

Simulations of the long-term (slow) dynamics with two buffers are shown in Fig. 7. The initial values for root and shoot biomass correspond to the (fixed) values for those state variables in the phase plane for the fast dynamics in Fig. 6. As the slow dynamics progress, the phase plane of the fast dynamics would change. The long-term simulations are started with two different choices for the initial values for the fast variables. The simulations demonstrate that the initialization of the fast variables can not only influence a short initial transient of the fast variables, but also influence long-term behavior by determining whether the system starts either with only the root growing or with only the shoot growing.

### 3.4 Reducing the number of additional state variables

The bottom row of Fig. 5 shows how the root–shoot model can be uniquely defined with a single buffer for the nitrogen shared by the root or the carbon shared by the shoot. The loops in the model are interrupted by either adding a differential equation for $\rho _N$ or for $\rho _C$.

We can create an adjacency matrix that shows which fluxes are directly connected (each row showing how the flux indicated on the left depends on the other fluxes). The matrix notation can be used to apply graph theory algorithms to identify whether a graph for the model fluxes is cyclic (and thus does not define the model uniquely) and which choices for adding buffers to the model result in an acyclic graph (and thus defines the model uniquely).

Without additional state variables, the graph for the fast dynamics is cyclic (top row of Fig. 5). Its adjacency matrix is

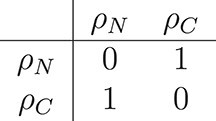



Adding state variables for the nitrogen and/or carbon shared makes the graph acyclic. Adding state variables for both surplus sharing fluxes (middle row of Fig. 5) results in the trivial adjacency matrix

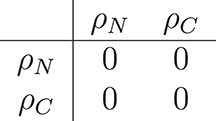



Adding a state variable for the nitrogen sharing flux (bottom row, left of Fig. 5) turns the adjacency matrix into
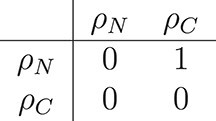


Adding a state variable for the carbon sharing flux (bottom row, right of Fig. 5), turns the adjacency matrix into
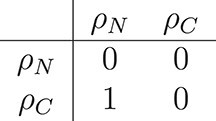


As seen in Fig. 5, any of the last three matrices describe an acyclic network that defines the model dynamics uniquely. Adding state variables for either of the surplus sharing fluxes is sufficient to define the model because it allows to calculate the other surplus sharing flux. Thus, any of the choices for which surplus to buffer completes the model description and allows to simulate the system.

## 4 The coral symbiont–host model

**Figure 8:**
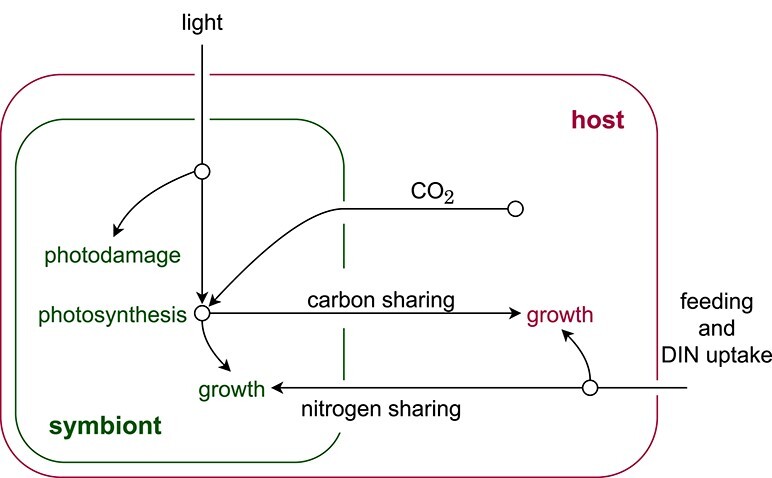
A sketch of the coral model by Cunning *et al.* (2017). The symbiont fixes carbon via photosynthesis and shares it with the host. In the other direction, the host shares nitrogen and CO$_2$ with the symbiont.

The model by Cunning *et al.* (2017) describes the dynamics of corals and their symbiotic algae. Similarly to the root–shoot model, the coral host shares nitrogen and the symbiont shares fixed carbon with their partners. A sketch of the model is shown in Fig. 8. The model details are stated in Appendix D.

### 4.1 Fast and slow dynamics

Many of the auxiliary variables (fluxes) in this model depend on other fluxes in the model. As in the root–shoot model, this makes it possible that multiple values for the fluxes satisfy the model equations for given $S$ and $H$ values.

Figure 10 shows solutions for the fluxes as functions of the symbiont–host ratio $S/H$, together with the corresponding growth rates of the symbiont and the host. The different solutions correspond to a ‘functional’ symbiosis (blue), a ‘dysfunctional’ symbiosis (orange) and an unstable equilibrium between those two states (green). In the functional symbiosis, photosynthesis, carbon shared by the symbiont and CO$_2$ available to the symbiont are high, while nitrogen shared by the host and photodamage of the symbiont are low. When the $S/H$ ratio is at its corresponding equilibrium (when $dS/dt/S - dH/dt/H = 0$), this state has a positive growth rate of symbiont and hosts ($dS/dt/S$ and $dH/dt/H$). The dysfunctional symbiosis describes the opposite case, including negative growth rates at the corresponding equilibrium $S/H$ ratio.

**Fig. 9:**
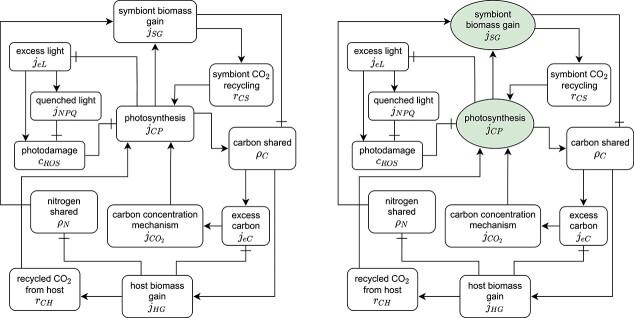
The fluxes in the coral model that form cycles. Normal arrowheads indicate a positive effect; blocked connector endings indicate a negative effect. Boxes indicate direct algebraic equations; green ovals indicate additional state variables that buffer in- and outputs. Left side: the model without additional state variables. Right side: the model with additional state variables for $j_{CP}$ and $j_{SG}$. The two additional state variables represent one of the choices that interrupt all cycles in the graph.

**Fig. 10:**
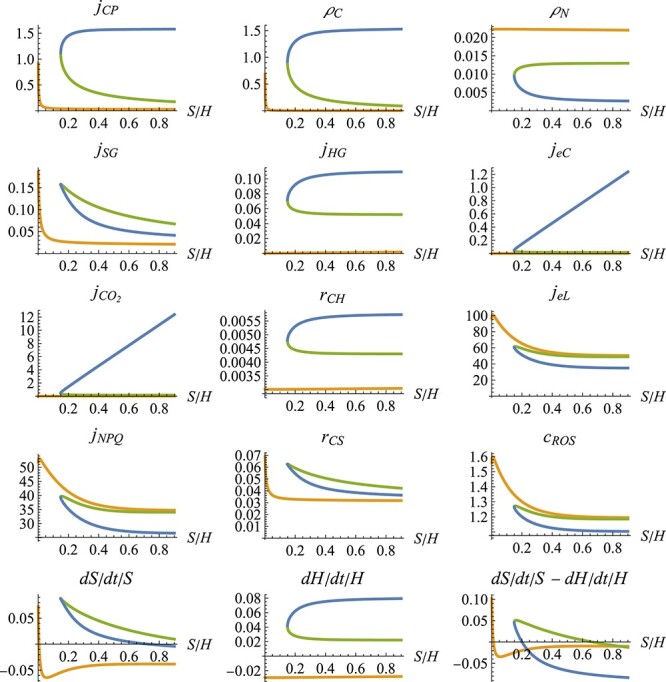
Different combinations of fluxes that satisfy the equations of the coral model in dependence of the symbiont–host ratio $S/H$. The colors indicate which equilibrium branches belong together. Parameter values as in Table 2 in Cunning *et al.* (2017), with additionally $L=30$, $N=1.5 \times 10^{-6}$ and $X=0$.

### 4.2 Adding state variables to the model to obtain a uniquely defined system and simulate the model

The model can be completed by adding state variables for all 12 fluxes involved in loops (the variables in the network shown in Fig. 9). Supplementary Fig. 1 shows the minimal combinations of buffers needed to complete the system. As all code, the implementation for finding those combinations is available online. The plots in the supplementary material show that the system needs at least two buffers. All two-buffer combinations include $j_{CP}$ as one of the additional state variables, and either $j_{HG}$, $j_{SG}$, $\rho _C$ or $\rho _N$ as the second additional state variable.

For the coral model, the fast state variables do not have an obvious mechanistic interpretation because of the complex interactions between the auxiliary variables. One could change the model formulation slightly to accommodate for buffers with clear mechanistic interpretations, as worked out for the root–shoot model. This could be done by choosing fluxes to buffer that represent transfer of substances (such as with our current choices the photosynthesis rate $j_{CP}$, but not the symbiont growth rate $j_{SG}$). However, working out the details is out of scope for this paper. For the present analysis, we introduced the fast state variables just as a numerical method to track solutions of the auxiliary network.

### 4.3 Initialization of the auxiliary variables

**Figure 11:**
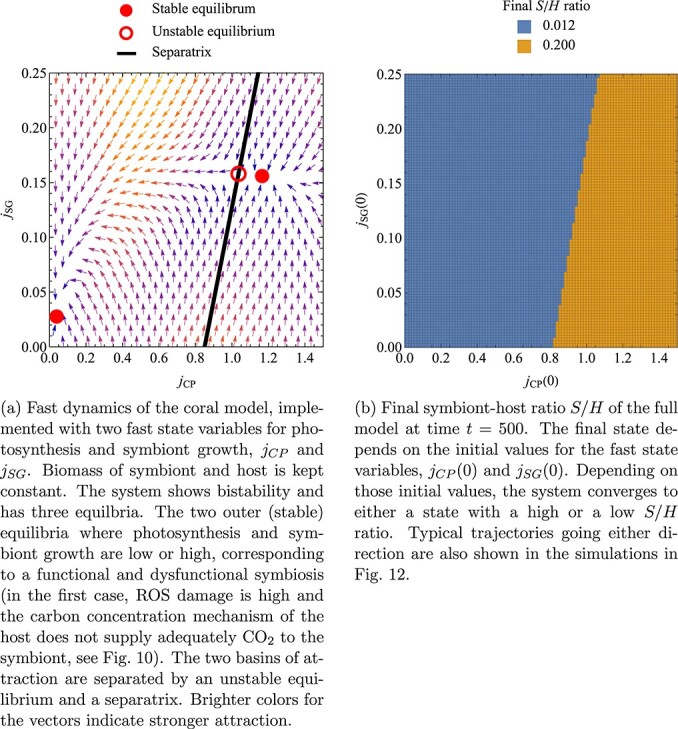
The bistability of the fast dynamics in the coral model can lead to different outcomes of the slow dynamics. Parameter values as in Fig. 10. Initial symbiont–host ratio is $S(0)/H(0)=0.15$. Numerical parameters for the additional state variables are $\lambda _{j_{CP}} = 10$ and $\lambda _{j_{SG}} = 10$.

**Figure 12:**
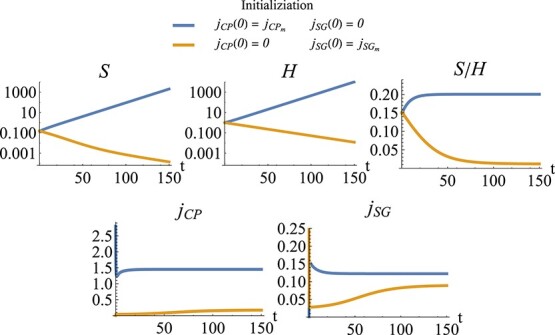
Simulation of the coral model, implemented with fast state variables for photosynthesis $j_{CP}$ and symbiont growth $j_{SG}$. From left to right and top to bottom: symbiont biomass (log scale), host biomass (log scale), ratio of symbiont to host, photosynthesis and symbiont growth rate. Blue curves: starting with high photosynthesis, $j_{CP}(0)=j_{CP_m}$, and low symbiont growth, $j_{SG}(0)=0$, leads to a functional symbiosis with a high $S/H$ ratio and positive growth rates. Orange curves: starting with low photosynthesis, $j_{CP}(0)=0$, and high symbiont growth, $j_{SG}(0)=j_{SG_m}$, leads to a dysfunctional symbiosis with a low $S/H$ ratio and negative growth rates. Parameter values as in Fig. 11. Initial symbiont to host ratio is $S(0)/H(0)=0.15$.

Our focus on carefully distinguishing and analyzing fast and slow dynamics is particularly important with this model, as the initial values of the chosen buffers can affect the model dynamics and the final state to which the system converges. The left side of Fig. 11 represents an initialization tool that shows how the initial values of the fast state variables can influence the dynamics of those state variables. The figure demonstrates the bistability of the fast dynamics, whereby the photosynthesis rate $j_{CP}$ and the symbiont growth rate $j_{SG}$ have been chosen as additional state variables. The fast dynamics can converge to either a state with high or low photosynthesis rate. The right side of Fig. 11 shows how this bistability of the fast dynamics in turn can influence the slow dynamics of the system (the change of biomass of symbiont and host). Depending on the initial photosynthesis rate, the system converges either to a functional symbiosis with a high $S/H$ ratio and positive growth rates or a dysfunctional symbiosis with a low $S/H$ ratio and negative growth rates. Figure 12 shows time trajectories of the same system, again demonstrating the bistability of the fast dynamics and the consequences on the slow system.

## 5 Discussion

DEB theory has been underutilized by ecological and evolutionary theorists modeling species interactions. It offers models of intermediate complexity that take account of thermodynamic and evolutionary constraints on the flows of energy and elemental matter at both ecological and evolutionary timescales (Jusup *et al.*, 2017). One possible reason for this neglect is that much ‘mainstream’ theory focuses on changes in qualitative properties of simplified dynamical systems. Full DEB models of species interactions are typically too equation- and parameter-rich to permit insightful qualitative analysis. To bridge this gap, many studies reduce the number of state variables, e.g. Chapter 9 of Kooijman (2010). A few studies archive further simplification by appealing to timescale separation, e.g. Poggiale *et al.* (2020). In similar spirit, we have here analyzed the dynamics arising from timescale separation in symbiotic interactions that involve shared metabolic products.

DEB models with timescale separation are of particular interest for modeling species interactions because they maintain the detailed physiological dynamics of single-organism DEB models while keeping model complexity low by assuming that physiological processes proceed faster than ecological dynamics. In published DEB-inspired models, timescale separation has been implemented by setting up differential equations for state variables that are solved simultaneously with algebraic equations. These algebraic equations can, in principle, be derived from underlying fast biochemical processes that equilibrate virtually instantaneously, but the details for those fast processes are often not explicitly provided in the model description. If these algebraic equations have a unique solution, simulating and analyzing such models is straightforward because the algebraic equations, together with the differential equations, unambiguously define the model dynamics. However, when the algebraic equations have multiple (or no) solutions, this is not true and the model dynamics are not defined uniquely anymore. In the present work, we showed how such ambiguity can be resolved by hypothesizing explicit fast dynamics that approach the solutions of the algebraic equations in the model specification. We emphasized that this type of ambiguity in the model definition is not only a technical mathematical issue since the initialization of the fast variables can impact long-term dynamics of the model system.

When setting up bioenergetic models that involve networks of fast processes, one often realizes quickly that such models can easily become complex and difficult to analyze. To address this issue, we offer a recipe for reducing the complexity of the underlying fast networks. The recipe is based on choosing a few auxiliary variables to be tracked directly through differential equations and evaluating the remaining quantities explicitly from these state variables. Biologically, this corresponds to assuming that the fast dynamic processes themselves act on different timescales: fast dynamics that are described through state variables and very fast dynamics that are assumed to equilibrate immediately and can be calculated using the rest of the system. Reducing the dimensionality of the system in this way can simplify numerical and analytical analysis. This simplification is especially useful for more complex models, such as our example for the symbiosis between corals and endosymbiotic algae (Cunning *et al.*, 2017), or even more for the extension of this model with multiple symbionts (Brown *et al.* 2022).

We demonstrate these approaches for two published models: one for the growth dynamics of the root and the shoot of a plant (Ledder *et al.*, 2020) and one for the growth dynamics of corals and their endosymbiotic algae (Cunning *et al.*, 2017). We show how these models can be simulated and how their analysis can be simplified by reducing their dimensionality. Both examples illustrate how the choice of initial conditions can affect a system’s state through cyclic feedbacks. In the example for the root and the shoot of a plant, an initially high carbon transfer triggers the system to start with the root growing, while an initially high nitrogen transfer triggers the system to start with the shoot growing. Similarly, in the coral example, the rate at which the symbiont shares carbon with its host turns out to be a particularly influential node in the fast network. Starting the system with a low photosynthesis rate can lead to a negative feedback loop, involving reduced activity of the host’s carbon concentration mechanism that provides CO$_2$ to the symbiont. The decreased CO$_2$ transfer in turn additionally reduces the rate at which the symbiont shares carbon with the host. Eventually, this cycle leads to a breakdown of the symbiosis. In this example, the bistability of the fast dynamics can affect the final state of the slow dynamics, i.e. the initialization of the fast network determines whether the system settles at a functional symbiosis characterized by a high symbiont to host ratio and positive growth rates, or whether it settles at a dysfunctional symbiosis characterized by a low symbiont to host ratio (bleached corals) and negative growth rates.

The bleaching of coral reefs is just one example of how understanding the breakdown of a symbiosis is critical for conservation. DEB models of ecological interactions, analyzed with our methods for simulating and simplifying the fast dynamics, could be useful in similar contexts. Such other examples where our approach could be used to study symbiosis include situations where abiotic changes driven by human activity (such as rising temperatures) can decouple the growth rates of hosts and their symbionts. This can impact animal growth (Greenspan *et al.*, 2020), the digestive system of humans (Carding *et al.*, 2015), the cycling of organic matter by the microbiome of marine macroalgae (Minich *et al.*, 2018) and the drought resistance of plants that harbor ectomycorrhizal fungi (Sapes *et al.*, 2021).

The scenarios described above can be all interpreted as bioenergetic systems. However, these are not the only type of systems where complex fast dynamics can have alternative equilibrium states. For example, alternative states of fast state variables have been reported in models that couple evolutionary and ecological dynamics (Brown & Akçay, 2019, Geritz *et al.*, 2002, Lehtinen & Geritz, 2019), in strictly ecological models (Rinaldi & Muratori, 1992) and in neurobiological models (Kurikawa & Kaneko, 2021, Wernecke *et al.*, 2018). In these examples, fast dynamics were either simulated directly, or the models were analyzed without simulating the full coupled system of fast and slow dynamics. These examples reflect that models from different areas could warrant approaches similar to those described in the present work, which allow reduction of the dimension of the state space and approximation of fast dynamics efficiently for simulations.

We end as we started—by emphasizing the unrealized potential for new DEB theory involving the exchange and interaction of metabolic products. Challenging areas include the ecology and evolution of the microbiome and its interaction with animal hosts, the evolution of mixotrophy, the emergence of microscale microbial consortia in aquatic systems—and of course there are many more. These and other fast processes may often drive population and community responses to changing environments, the core issue for conservation biology. Systematizing methodology for timescale separation in the absence of empirical data at all timescales will be essential in such work; we see this paper as a contribution to this effort.

## 6 Supplementary material

Supplementary Fig. 1: https://github.com/ferdi-p/time-scale-separation/blob/main/supplementary-figures/Supplementary-Fig-1-coral-model-buffer-combinations.pdf?raw=true

All code for the models has been implemented in Wolfram Mathematica 12.2 (Wolfram Research, 2021). The code is freely available at https://github.com/ferdi-p/time-scale-separation/tree/main/code.

## 7 Data availability

No new data were generated or analysed in support of this research.

## A Tikhonov’s theorem

Tikhonov’s theorem (Klonowski, 1983, Tikhonov, 1952) states that under certain conditions the fast-slow system

(A.1) \begin{align*}& \begin{aligned} \frac{d{{X}\kern-6pt^{{}^{{}^{\rightarrow}}}}}{dt} &= f({{X}\kern-6pt^{{}^{{}^{\rightarrow}}}},{{Z}\kern-6pt^{{}^{{}^{\rightarrow}}}},t)\\ \frac{d{{Z}\kern-6pt^{{}^{{}^{\rightarrow}}}}}{dt} &= \gamma h({{X}\kern-6pt^{{}^{{}^{\rightarrow}}}},{{Z}\kern-6pt^{{}^{{}^{\rightarrow}}}},t) \end{aligned} \end{align*}

converges for $\gamma \rightarrow \infty $ to the form

(A.2) \begin{align*}& \begin{aligned} \frac{d{{X}\kern-6pt^{{}^{{}^{\rightarrow}}}}}{dt} &= f({{X}\kern-6pt^{{}^{{}^{\rightarrow}}}},\phi({{X}\kern-6pt^{{}^{{}^{\rightarrow}}}},t),t) \end{aligned} \end{align*}

as long as the point $({{X}\kern-6pt^{{}^{{}^{\rightarrow}}}},t)$ does not leave a certain region $D$ of the state space.

The conditions are as follows:

1. ${{Z}\kern-6pt^{{}^{{}^{\rightarrow}}}}=\phi ({{X}\kern-6pt^{{}^{{}^{\rightarrow}}}},t)$ is an isolated solution of $h({{X}\kern-6pt^{{}^{{}^{\rightarrow}}}},{{Z}\kern-6pt^{{}^{{}^{\rightarrow}}}},t)=0$ whose points are locally (asymptotically) stable equilibrium points of the adjoined system
  (A.3) \begin{align*}& \begin{aligned} \frac{d{{Z}\kern-6pt^{{}^{{}^{\rightarrow}}}}}{dt} &= h({{X}\kern-6pt^{{}^{{}^{\rightarrow}}}},{{Z}\kern-6pt^{{}^{{}^{\rightarrow}}}},t) \end{aligned} \end{align*}
  (${{X}\kern-6pt^{{}^{{}^{\rightarrow}}}}$, $t$ being considered here as parameters, the point $({{X}\kern-6pt^{{}^{{}^{\rightarrow}}}},t)$ belonging to a bounded open region $D$).
2. The initial conditions $({{X}\kern-6pt^{{}^{{}^{\rightarrow}}}}^0,{{Z}\kern-6pt^{{}^{{}^{\rightarrow}}}}^0,t^0)$ are such that the solution ${{Z}\kern-6pt^{{}^{{}^{\rightarrow}}}}(\tau )$ of the adjoined system
  (A.4) \begin{align*}& \begin{aligned} \frac{d{{Z}\kern-6pt^{{}^{{}^{\rightarrow}}}}}{d\tau} &= h({{X}\kern-6pt^{{}^{{}^{\rightarrow}}}}^0,{{Z}\kern-6pt^{{}^{{}^{\rightarrow}}}},t^0)\\ {{Z}\kern-6pt^{{}^{{}^{\rightarrow}}}}(0) &= {{Z}\kern-6pt^{{}^{{}^{\rightarrow}}}}^0 \end{aligned} \end{align*}
  satisfies $\lim _{\tau \to \infty } {{Z}\kern-6pt^{{}^{{}^{\rightarrow}}}}(\tau ) = \phi ({{X}\kern-6pt^{{}^{{}^{\rightarrow}}}}^0,t^0)$.
3. ${{X}\kern-6pt^{{}^{{}^{\rightarrow}}}}$ , $f$ are $n$-vectors, ${{Z}\kern-6pt^{{}^{{}^{\rightarrow}}}}$, $h$$m$-vectors and $f$, $h$ satisfy suitable regularity assumptions.

## B Details to differential equations for fast and slow dynamics

To apply this theorem to our type of system, we assume that the algebraic equation for ${{Z}\kern-6pt^{{}^{{}^{\rightarrow}}}}$ can be defined through the equilibrium states of a differential equation on a fast timescale (even when those equations are not explicitly stated in the model description). The form of the differential equation is generally

(B.1) \begin{align*}& \begin{aligned} \frac{d{{Z}\kern-6pt^{{}^{{}^{\rightarrow}}}}}{dt} &= \gamma h({{X}\kern-6pt^{{}^{{}^{\rightarrow}}}},{{Z}\kern-6pt^{{}^{{}^{\rightarrow}}}}). \end{aligned} \end{align*}

Here $\gamma $ is a parameter that determines the speed of the system. Tikhonov’s theorem loosely tells us that the slow variables converge for $\gamma \rightarrow \infty $ to the form

(B.2) \begin{align*}& \begin{aligned} \frac{d{{X}\kern-6pt^{{}^{{}^{\rightarrow}}}}}{dt} &= f({{X}\kern-6pt^{{}^{{}^{\rightarrow}}}},\phi({{X}\kern-6pt^{{}^{{}^{\rightarrow}}}})), \end{aligned} \end{align*}

where ${{Z}\kern-6pt^{{}^{{}^{\rightarrow}}}}=\phi ({{X}\kern-6pt^{{}^{{}^{\rightarrow}}}})$ is an isolated solution of $h({{X}\kern-6pt^{{}^{{}^{\rightarrow}}}},{{Z}\kern-6pt^{{}^{{}^{\rightarrow}}}})=0$ whose points are locally (asymptotically) stable equilibrium points of the system (B.1). The equilibria correspond to the solutions of ${{Z}\kern-6pt^{{}^{{}^{\rightarrow}}}} = g ( {{X}\kern-6pt^{{}^{{}^{\rightarrow}}}}, {{Z}\kern-6pt^{{}^{{}^{\rightarrow}}}} )$. In principle, the only formal information we have on $h$ is that it must satisfy the equilibria conditions, i.e.

(B.3) \begin{align*}& \begin{aligned} {{Z}\kern-6pt^{{}^{{}^{\rightarrow}}}} = g ( {{X}\kern-6pt^{{}^{{}^{\rightarrow}}}}, {{Z}\kern-6pt^{{}^{{}^{\rightarrow}}}} ) \Longleftrightarrow h({{X}\kern-6pt^{{}^{{}^{\rightarrow}}}},{{Z}\kern-6pt^{{}^{{}^{\rightarrow}}}})=0. \end{aligned} \end{align*}

In the absence of more information, we have to add assumptions to specify the model dynamics. It seems generally reasonable to assume that each component $Z_i$ of the network is attracted to its ‘target’ $g_i ( {{X}\kern-6pt^{{}^{{}^{\rightarrow}}}}, {{Z}\kern-6pt^{{}^{{}^{\rightarrow}}}} )$, i.e. the sign of the time derivative $h_i$ is the same as the sign of the difference between target and state,

(B.4) \begin{align*}& \begin{aligned} \text{sign}(h_i({{X}\kern-6pt^{{}^{{}^{\rightarrow}}}},{{Z}\kern-6pt^{{}^{{}^{\rightarrow}}}})) =\text{sign}( g_i ( {{X}\kern-6pt^{{}^{{}^{\rightarrow}}}}, {{Z}\kern-6pt^{{}^{{}^{\rightarrow}}}} ) - Z_i ). \end{aligned} \end{align*}

Qualitatively, this behavior can be captured by linearization, i.e.

(B.5) \begin{align*}& \begin{aligned} h_i({{X}\kern-6pt^{{}^{{}^{\rightarrow}}}},{{Z}\kern-6pt^{{}^{{}^{\rightarrow}}}}) &= c_i ( g_i ( {{X}\kern-6pt^{{}^{{}^{\rightarrow}}}}, {{Z}\kern-6pt^{{}^{{}^{\rightarrow}}}} ) - Z_i ), \end{aligned} \end{align*}

where $c_i>0$ are constants that determine the (relative) speed with which each part of the network approaches its target. Choosing $\lambda _i=\gamma c_i$, this brings us to equation (4)

(B.6) \begin{align*}& \begin{aligned} \frac{d Z_i}{dt} &= \lambda_i ( g_i({{X}\kern-6pt^{{}^{{}^{\rightarrow}}}},{{Z}\kern-6pt^{{}^{{}^{\rightarrow}}}}) - Z_i ). \end{aligned} \end{align*}

Note that the different $\lambda _i$ are generally not specified by the model (and they can in principle also depend on the state of the system, i.e. which equilibrium is in the vicinity of the auxiliary network). When applying the method to a new model, it can be useful to try different large values for the $\lambda _i$ and check convergence.

It can be useful to vectorize the model. Let

(B.7) \begin{align*}& \begin{aligned} \Lambda= \left(\begin{array}{cccc} \lambda_1 & 0 & \dots & 0 \\ 0 & \lambda_2 & \dots & 0 \\ \vdots & \vdots & \ddots & \vdots \\ 0 & 0 & \dots \end{array}\right) \end{aligned} \end{align*}

so that

(B.8) \begin{align*}& \begin{aligned} \frac{d {{Z}\kern-6pt^{{}^{{}^{\rightarrow}}}}}{dt} &= \Lambda ( g({{X}\kern-6pt^{{}^{{}^{\rightarrow}}}},{{Z}\kern-6pt^{{}^{{}^{\rightarrow}}}}) - {{Z}\kern-6pt^{{}^{{}^{\rightarrow}}}} ). \end{aligned} \end{align*}

In absence of any other information, a simple choice can be to choose all $\lambda _i$ equal and reasonably big.

The scheme we propose is asymptotically a generalization of the discrete time scheme implemented in earlier work (Cunning *et al.*, 2017), when all $\lambda _i = \lambda $ and the time step $\Delta t = 1/\lambda $ is small. This can be seen by expressing equation (4) as

(B.9) \begin{align*}& \begin{aligned} \frac{{{Z}\kern-6pt^{{}^{{}^{\rightarrow}}}}(t+\Delta t) - {{Z}\kern-6pt^{{}^{{}^{\rightarrow}}}}(t)}{\Delta t} &= \frac{g({{X}\kern-6pt^{{}^{{}^{\rightarrow}}}}(t),{{Z}\kern-6pt^{{}^{{}^{\rightarrow}}}}(t)) - {{Z}\kern-6pt^{{}^{{}^{\rightarrow}}}}(t)}{\Delta t} \end{aligned} \end{align*}

and solving for ${{Z}\kern-6pt^{{}^{{}^{\rightarrow}}}}(t+\Delta t)$ so that

(B.10) \begin{align*}& \begin{aligned} {{Z}\kern-6pt^{{}^{{}^{\rightarrow}}}}(t+\Delta t) &= g({{X}\kern-6pt^{{}^{{}^{\rightarrow}}}}(t),{{Z}\kern-6pt^{{}^{{}^{\rightarrow}}}}(t)). \end{aligned} \end{align*}

## C Dimensionality reduction

Divide the complete set of auxiliary variables into two sets, $Z_A$ and $Z_B,$

(C.1) \begin{align*}& \begin{aligned} {{Z}\kern-6pt^{{}^{{}^{\rightarrow}}}} &= {{Z}\kern-6pt^{{}^{{}^{\rightarrow}}}}_A \cup {{Z}\kern-6pt^{{}^{{}^{\rightarrow}}}}_B \end{aligned} \end{align*}

(where $\cup $ refers to ‘combining the two vectors and rearranging them in the original order’) and

(C.2) \begin{align*}& \begin{aligned} g({{Z}\kern-6pt^{{}^{{}^{\rightarrow}}}}) &= g_A( {{X}\kern-6pt^{{}^{{}^{\rightarrow}}}},{{Z}\kern-6pt^{{}^{{}^{\rightarrow}}}}_A \cup {{Z}\kern-6pt^{{}^{{}^{\rightarrow}}}}_B ) \cup g_B( {{X}\kern-6pt^{{}^{{}^{\rightarrow}}}},{{Z}\kern-6pt^{{}^{{}^{\rightarrow}}}}_A) \end{aligned} \end{align*}

then it is enough to set up dynamics for ${{Z}\kern-6pt^{{}^{{}^{\rightarrow}}}}_A$ and derive $ {{Z}\kern-6pt^{{}^{{}^{\rightarrow}}}}_B$ from it, which completes the network. That is, ${{Z}\kern-6pt^{{}^{{}^{\rightarrow}}}}_A$ is described by a differential equation

(C.3) \begin{align*}& \begin{aligned} \frac{d {{Z}\kern-6pt^{{}^{{}^{\rightarrow}}}}_A}{dt} &= \Lambda_A ( g_A({{X}\kern-6pt^{{}^{{}^{\rightarrow}}}},{{Z}\kern-6pt^{{}^{{}^{\rightarrow}}}}_A \cup {{Z}\kern-6pt^{{}^{{}^{\rightarrow}}}}_B ) - {{Z}\kern-6pt^{{}^{{}^{\rightarrow}}}}_A ), \end{aligned} \end{align*}

where $\Lambda _A$ is a diagonal matrix that contains the numerical parameters $\lambda _i$ for how fast the different components of ${{Z}\kern-6pt^{{}^{{}^{\rightarrow}}}}_A$ approach their targets. The remainder of the network, ${{Z}\kern-6pt^{{}^{{}^{\rightarrow}}}}_B$, can then be calculated directly:

(C.4) \begin{align*}& \begin{aligned} {{Z}\kern-6pt^{{}^{{}^{\rightarrow}}}}_B &= g_B ( {{X}\kern-6pt^{{}^{{}^{\rightarrow}}}}, {{Z}\kern-6pt^{{}^{{}^{\rightarrow}}}}_A ). \end{aligned} \end{align*}

When the fast dynamics are at their equilibrium, any choice for which auxiliary variables to track as state variables satisfies the model specification. While in our experience the choices make little difference, they can principally differ in their dynamics. When setting up a new model, it can be useful to try different combinations of fast state variables and compare simulations.

## D Coral model formulation

This section describes the details of the coral model by Cunning *et al.* (2017). The model tracks the biomass of symbiont $S$ and host $H$. Additionally, it tracks various fluxes within and between the organisms, as shown in Table D1. The model parameters are shown in Table D2. Sticking with the notations of the original papers, the coral model formulation differs slightly from the root-shoot model formulation in that for the coral model the fluxes are measured per biomass (either of the host or the symbiont, depending on where the flux is originating from) while in the root–shoot model the fluxes describe the total values (i.e. already accounting for the biomass of the organ where the flux is originating from). Both models could be formulated in either way, but for consistency with the original papers we stick with the formulations used there. We also fixed two obvious typos in the original formulation of the coral model, changing slightly the equations for $j_{HG}$ and $\rho _N$.

**Table D1:** Coral model state variables and fluxes.

State variables		
Symbol	Description	Units
$S$	Symbiont biomass	mol C
$H$	Host biomass	mol C
Fluxes and other auxiliary variables		
Symbol	Description	Units
$j_X$	Prey assimilation (feeding) rate	mol C mol C$^{-1}$ d$^{-1}$
$j_N$	Nitrogen uptake rate	mol N mol C$^{-1}$ d$^{-1}$
$j_{HG}$	Host biomass formation rate	mol C mol C$^{-1}$ d$^{-1}$
$j_{HT}$	Host biomass turnover rate	mol C mol C$^{-1}$ d$^{-1}$
$r_{NH}$	Recycled nitrogen from host turnover	mol N mol C$^{-1}$ d$^{-1}$
$\rho _N$	Nitrogen shared with the symbiont	mol N mol C$^{-1}$ d$^{-1}$
$j_{eC}$	Excess carbon used to activate host CCMs	mol C mol C$^{-1}$ d$^{-1}$
$j_{CO_2}$	$CO_{2}$ input to photosynthesis	mol C mol C$^{-1}$ d$^{-1}$
$A$	Light amplification factor	-
$j_{L}$	Light absorption rate for symbiont	mol photons mol C$^{-1}$ d$^{-1}$
$r_{CH}$	Recycled $CO_{2}$ from host	mol C mol C$^{-1}$ d$^{-1}$
$r_{CS}$	Recycled $CO_{2}$ from symbiont	mol C mol C$^{-1}$ d$^{-1}$
$j_{CP}$	Photosynthesis rate of symbiont	mol C mol C$^{-1}$ d$^{-1}$
$j_{eL}$	Light energy in excess of photochemistry for symbiont	mol photons mol C$^{-1}$ d$^{-1}$
$j_{NPQ}$	Total capacity of NPQ of symbiont	mol photons mol C$^{-1}$ d$^{-1}$
$c_{ROS}$	ROS production proportional to baseline for symbiont	-
$r_{NS}$	Recycled from turnover of symbiont	mol N mol C$^{-1}$ d$^{-1}$
$j_{SG}$	Symbiont biomass formation rate	mol C mol C$^{-1}$ d$^{-1}$
$\rho _{C}$	Fixed carbon symbiont shares with host	mol C mol C$^{-1}$ d$^{-1}$
$j_{ST}$	Symbiont biomass turnover rate	mol C mol C$^{-1}$ d$^{-1}$

**Table D2:** Coral model parameters (for typical values, see Cunning *et al.*, 2017)

Environment	
Symbol	Description	Units
$L$	Light intensity	mol photons m$^{-2}$ d$^{-2}$
$X$	Prey availability	mol C L$^{-1}$
$N$	DIN concentration (dissolved inorganic nitrogen)	mol N L$^{-1}$
Parameters		
Symbol	Description	Units
$n_{NH}$	N:C molar ratio in host biomass	mol N mol C$^{-1}$
$n_{NS}$	N:C molar ratio in symbiont biomass	mol N mol C$^{-1}$
$n_{NX}$	N:C molar ratio in prey biomass	mol N mol C$^{-1}$
$j_{HT}^0$	Maintenance rate of host biomass	mol C mol C$^{-1}$ d$^{-1}$
$j_{ST}^0$	Maintenance rate of symbiont biomass	mol C mol C$^{-1}$ d$^{-1}$
$\sigma _{NH}$	Proportion N turnover recycled in host	mol N mol N$^{-1}$
$\sigma _{CH}$	Proportion host metabolic $CO_{2}$ recycled to photosynthesis	mol C mol C$^{-1}$
$\sigma _{NS}$	Proportion N turnover recycled in symbiont	mol N mol N$^{-1}$
$\sigma _{CS}$	Proportion symbiont metabolic $CO_{2}$ recycled to photosynthesis	mol C mol C$^{-1}$
$j_{X_m}$	Maximum prey assimilation rate from host feeding	mol C mol C$^{-1}$ d$^{-1}$
$K_X$	Half-saturation constant for prey assimilation	mol C L$^{-1}$
$j_{N_m}$	Maximum host DIN uptake rate	mol N mol C$^{-1}$ d$^{-1}$
$K_N$	Half-saturation constant for host DIN uptake	mol N L$^{-1}$
$k_{CO_{2}}$	Efficacy of $CO_{2}$ delivery to photosynthesis by host CCMs	mol C mol C$^{-1}$
$j_{HG_m}$	Maximum specific growth rate of host	mol C mol C$^{-1}$ d$^{-1}$
$y_{CL}$	Quantum yield of photosynthesis for symbiont	mol C mol photons$^{-1}$
$y_C$	Yield of biomass formation from carbon	-
$\bar {a}^*$	Effective light-absorbing cross-section of symbiont	m$^2$ mol C$^{-1}$
$k_{NPQ}$	NPQ capacity of symbiont	mol photons mol C$^{-1}$ d$^{-1}$
$k_{ROS}$	Excess photon energy that doubles ROS production in symbiont, relative to baseline levels	mol photons mol C$^{-1}$ d$^{-1}$
$j_{CP_m}$	Maximum specific photosynthesis rate of symbiont	mol C mol C$^{-1}$ d$^{-1}$
$j_{SG_m}$	Maximum specific growth rate of symbiont	mol C mol C$^{-1}$ d$^{-1}$
$b$	Scaling parameter for bleaching response, symbiont	-

For convenience, we define notations for the functional responses used. For the uptake of a single substrate $x$ the Michaelis–Menten-type SU is defined by

(D.1) \begin{align*}& \begin{aligned} f(max,k;x) &= max \frac{x}{k+x}. \end{aligned} \end{align*}

To combine two substrates, $x$ and $y$, the model uses a ‘parallel complementary’ SU (Kooijman, 2010)

(D.2) \begin{align*}& \begin{aligned} F(max;x,y) &= \frac{1}{\frac{1}{max}+\frac{1}{x}+\frac{1}{y}-\frac{1}{x+y}}. \end{aligned} \end{align*}

The change of biomass of symbiont and host are given by terms for growth and for biomass loss (including turnover)

(D.3) \begin{align*}& \begin{aligned} \frac{dS}{dt} &= j_{SG} - j_{ST}\\ \frac{dH}{dt} &= j_{HG} - j_{HT} \end{aligned}. \end{align*}

The fluxes and other auxiliary variables are defined implicitly at their fast equilibrium states by

(D.4) \begin{align*}& \begin{aligned} j_X &= f(j_{X_m},K_X; X)\\ j_N &= f(j_{N_m},K_N; N)\\ j_{HG} &= F \left( j_{HGm}; y_C \left(\rho_C \frac{S}{H}\!+\! j_X\right), \left(j_N \!+\! n_{NX} j_X \!+\!r_{NH} \right) n_{NH}^{-1} \right)\\ j_{HT} &= j_{HT}^0\\ r_{NH} &= \sigma_{NH} n_{NH} j_{HT}
\end{aligned} \end{align*}

(D.4)
\begin{align*}& \begin{aligned} \rho_N &= j_N + n_{NX} j_X + r_{NH} - n_{NH} j_{HG}\\ j_{eC} &= j_X + \rho_C \frac{S}{H} - j_{HG} y_C^{-1}\\ j_{CO_2} &= k_{CO_2} j_{eC}\\ A &= 1.26 + 1.39 e^{- 6.48 \frac{S}{H}}\\ j_L &= A L \bar{a}^*\\ r_{CH} &= \sigma_{CH} ( j_{HT} + (1-y_C) j_{HG} y_C^{-1}\\
r_{CS} &= \sigma_{CS} (j_{ST}^0 + (1-y_C)j_{SG} y_C^{-1})
\end{aligned} \end{align*}

(D.4)
\begin{align*}& \begin{aligned} j_{CP} &= F \left( j_{CPm}; y_{CL} j_L, \left(j_{CO_2} + r_{CH} \right) \frac{H}{S} + r_{CS} \right) c_{\text{ROS}}^{-1}\\ j_{eL} &= j_L - j_{CP} y_{CL}^{-1}\\ j_{NPQ} &= \left( k_{NPQ}^{-1} + j_{eL}^{-1} \right)^{-1}\\ c_{ROS} &= 1 + \frac{ \max (j_{eL} - j_{NPQ},0)}{k_{ROS}}\\ r_{NS} &= \sigma_{NS} n_{NS} j_{ST}^0\\ j_{SG} &= F \left( j_{SGm}; y_{C} j_{CP}, \left(\rho_N \frac{H}{S} + r_{NS} \right) n_{NS}^{-1} \right)\\ \rho_C &= j_{CP} - j_{SG} y_C^{-1}\\ j_{ST} &= j_{ST}^0 (1 + b ( c_{ROS} - 1 ) ). \end{aligned} \end{align*}

For details, see Cunning *et al.* (2017).

## References

[ref1] Brown A and Akçay E (2019) Evolution of transmission mode in conditional mutualisms with spatial variation in symbiont quality. Evolution, 73(2): 128–144. 30536933 10.1111/evo.13656

[ref2] Brown A. L. , Pfab F., Baxter E. C., Detmer A. R., Moeller H. V., Nisbet R. M. and Cunning R. (2022) Analysis of a mechanistic model of corals in association with multiple symbionts: Within-host competition and recovery from bleaching. Submitted for publication. 10.1093/conphys/coac066 PMC9558299 36247693

[ref3] Carding S , Verbeke K, Vipond D T, Corfe B M, Owen L J (2015) Dysbiosis of the gut microbiota in disease. Microbial ecology in health and disease, 26:26191. 25651997 10.3402/mehd.v26.26191 PMC4315779

[ref4] Cunning R , Muller EB, Gates RD, Nisbet RM (2017) A dynamic bioenergetic model for coral-symbiodinium symbioses and coral bleaching as an alternate stable state. J Theor Biol 431: 49–62. 28782552 10.1016/j.jtbi.2017.08.003

[ref5] Eynaud, Y., Nisbet, R. M., and Muller, E. B. (2011) Impact of excess and harmful radiation on energy budgets in scleractinian corals. Ecol Model, 222:1315–1322.

[ref6] Geritz, S. A., Gyllenberg, M., Jacobs, F. J., and Parvinen, K. (2002) Invasion dynamics and attractor inheritance. J Math Biol, 44:548–560. 12111102 10.1007/s002850100136

[ref7] Greenspan, S. E., Migliorini, G. H., Lyra, M. L., Pontes, M. R., Carvalho, T., Ribeiro, L. P., Moura-Campos, D., Haddad, C. F., Toledo, L. F., Romero, G. Q., Becker C. G. (2020) Warming drives ecological community changes linked to host-associated microbiome dysbiosis. Nat Clim Change 10:1057–1061.

[ref8] Jusup M , Sousa T, Domingos T, Labinac V, Marn N, Wang Z, Klanjšček T (2017) Physics of metabolic organization. Phys Life Rev 20: 1–39. 27720138 10.1016/j.plrev.2016.09.001

[ref9] Kearney, M. R. and Porter, W. P. (2020) NicheMapR–an R package for biophysical modelling: the ectotherm and dynamic energy budget models. Ecography 43:85–96.

[ref10] Klonowski, W. (1983) Simplifying principles for chemical and enzyme reaction kinetics. Biophys Chem 18:73–87. 6626688 10.1016/0301-4622(83)85001-7

[ref11] Kooi, B., Kuijper, L., and Kooijman, S. (2004) Consequences of symbiosis for food web dynamics. J Math Biol 49:227–271. 15293013 10.1007/s00285-003-0256-0

[ref12] Kooijman B (1993) Dynamic energy budgets in biological systems. In Theory and Applications in Ecotoxicology. Cambridge, United Kingdom: Cambridge University Press.

[ref13] Kooijman B (2010) Dynamic Energy Budget Theory for Metabolic Organisation. Cambridge University Press.

[ref14] Kooijman, S. (2001) Quantitative aspects of metabolic organization: a discussion of concepts. Phil Trans R Soc Lond B 356:331–349. 11316483 10.1098/rstb.2000.0771 PMC1088431

[ref15] Kooijman, S., Auger, P., Poggiale, J., and Kooi, B. (2003) Quantitative steps in symbiogenesis and the evolution of homeostasis. Biol Rev 78:435–463. 14558592 10.1017/s1464793102006127

[ref16] Kooijman, S. and Metz, J. (1984) On the dynamics of chemically stressed populations: the deduction of population consequences from effects on individuals. Ecotoxicol Environ Saf 8:254–274. 6734503 10.1016/0147-6513(84)90029-0

[ref17] Kurikawa T , Kaneko K (2021) Multiple-timescale neural networks: generation of history-dependent sequences and inference through autonomous bifurcations. Front Comput Neurosci 15. 10.3389/fncom.2021.743537 PMC8702558 34955798

[ref18] Lavaud, R., Filgueira, R., and Augustine, S. (2021) The role of dynamic energy budgets in conservation physiology. Conserv Phys Ther 9: coab083. 10.1093/conphys/coab083 PMC8545044 34707875

[ref19] Ledder, G., Russo, S. E., Muller, E. B., Peace, A., and Nisbet, R. M. (2020) Local control of resource allocation is sufficient to model optimal dynamics in syntrophic systems. Theor Ecol 13:481–501.

[ref20] Lehtinen SO , Geritz SA (2019) Cyclic prey evolution with cannibalistic predators. J Theor Biol 479: 1–13. 31265847 10.1016/j.jtbi.2019.06.025

[ref21] Minich, J. J., Morris, M. M., Brown, M., Doane, M., Edwards, M. S., Michael, T. P., and Dinsdale, E. A. (2018) Elevated temperature drives kelp microbiome dysbiosis, while elevated carbon dioxide induces water microbiome disruption. PLoS One 13:e0192772. 10.1371/journal.pone.0192772 PMC5825054 29474389

[ref22] Muller, E. B., Kooijman, S. A., Edmunds, P. J., Doyle, F. J., and Nisbet, R. M. (2009) Dynamic energy budgets in syntrophic symbiotic relationships between heterotrophic hosts and photoautotrophic symbionts. J Theor Biol 259:44–57. 19285512 10.1016/j.jtbi.2009.03.004

[ref23] Murphy, C. A., Nisbet, R. M., Antczak, P., Garcia-Reyero, N., Gergs, A., Lika, K., Mathews, T., Muller, E. B., Nacci, D., Peace, A., et al. (2018) Incorporating suborganismal processes into dynamic energy budget models for ecological risk assessment. Integr Environ Assess Manag 14:615–624. 29870141 10.1002/ieam.4063 PMC6643959

[ref24] Poggiale, J.-C., Aldebert, C., Girardot, B., and Kooi, B. W. (2020) Analysis of a predator–prey model with specific time scales: a geometrical approach proving the occurrence of canard solutions. J Math Biol 80:39–60. 30788562 10.1007/s00285-019-01337-4

[ref25] Rinaldi, S. and Muratori, S. (1992) Limit cycles in slow-fast forest-pest models. Theor Popul Biol 41:26–43.

[ref26] Sapes, G., Demaree, P., Lekberg, Y., and Sala, A. (2021) Plant carbohydrate depletion impairs water relations and spreads via ectomycorrhizal networks. New Phytol 229:3172–3183. 33280134 10.1111/nph.17134

[ref27] Schouten R , Vesk PA, Kearney MR (2020) Integrating dynamic plant growth models and microclimates for species distribution modelling. Ecol Model 435: 109262.

[ref28] Tikhonov, A. (1952) Systems of differential equations containing small parameters in the derivatives. Mat Sb Nov Ser 31:575–586.

[ref29] Wernecke, H., Sándor, B., and Gros, C. (2018) Attractor metadynamics in terms of target points in slow-fast systems: adiabatic versus symmetry protected flow in a recurrent neural network. J Phys Commun 2:095008.

[ref30] Wolfram Research (2021) Mathematica. Version 12.2.

